# Case of Small-Pox Subsequent to Vaccination

**Published:** 1811-05

**Authors:** James Barlow

**Affiliations:** No. 3, Little George-Street, Minories


*To the Editors of the Medical and Physical Journal.*

### *Case of Small-Pox subsequent to Vaccination.*

GENTLEMEN,

BY inserting the following Case of Small-pox subsequent to Vaccination, in your periodical Journal, you will oblige

Your obedient Servant,

JAMES BARLOW.

About eight years ago I inoculated with the same lancet, at the same time, two children belonging to Mr. T——, of Blackburn, Lancashire, with vaccine fluid taken from a subject on the seventh day after vaccination; the disease was communicated in both instances, and went through its usual course regularly and satisfactorily, leaving two distinct scars in both the arms of each child, which are visible at the present time.

After an interval of seven years from the time of vaccination, the above-mentioned children were exposed to the contagion arising from a person labouring under the confluent Small-pox, and soon after, the subject of this case was attacked with fever and sickness, and at the usual time after seizure, an eruption appeared all over the body, which unquestionably terminated in the Small-pox. The pustules were pretty numerous and distinct, and gradually filled with matter, which dried away in eight or ten days from the time of their appearance, leaving bluish marks on the cuticle. During the progress of the eruption, the child, at my request, was visited by Mr. Coultate, a practitioner in the town, who fully concurred with me in opinion respecting the identity of the disease.

It is far from my intention, by relating this case of small-pox subsequent to vaccination, to invalidate the latter disease, for it is the only instance of failure which I have witnessed; nevertheless, it may not be altogether improper to remark, that the advocates for cow-pox appear to me to have carried their prejudices against inoculation too far, as is evident from the perusal of the works of most authors who have written on vaccination that they have drawn their conclusions in favour of the utility of cow-pox over small-pox inoculation with too much precipitation, almost leaving small-pox inoculation out of the question. Reflecting on this circumstance, I am led to believe that if the faculty had encouraged both modes of inoculation agreeably to the inclination of the parents, a much greater number of children would would have undergone inoculation since the promulgation of the new disease by Dr. Jenner; consequently fewer individuals would have been exposed to the ravages of the casual small-pox than what appears from consulting the records of medical history as connected with vaccination; hence will appear the advantage of recommending both vaccination and inoculation, and thereby secure a greater number of children from one of the greatest evils to which the human race are so unavoidably exposed. Probably most advocates for vaccination have had in view the total extirmination of small-pox; such a plan, however extensively pursued in this country, promises but little towards producing the desired effect; and were it even possible to banish the disease from this island, we are still liable to be assailed anew from the continent with which we have free intercourse, from which will result the impossibility of totally rooting out this morbific disease from this country, unless a similar plan were at the same time attempted in every part of the known world; and it is to be doubted whether such a scheme, though successful for a period, would prove eventually permanent. I am aware that the sentiment I am about to advance, regarding the non-contagious effects of small-pox inoculation, will meet with an adverse opinion from the advocates for vaccination; nevertheless I am actuated to hazard this idea from motives of utility, and a conviction in my own mind, that infection, arising from small-pox inoculation, is a very rare occurrence; for in most instances where small-pox has been propagated from one individual to another, I have been able to trace its source to have arisen from that disease, and never from inoculation, unless the disease assumed the confluent character.

I remain, Gentlemen,

Your obedient Servant,

JAMES BARLOW.

*No. 3, Little George-Street, Minories.*

